# Presepsin and procalcitonin as predictors of sepsis based on the new Sepsis-3 definitions in obstructive acute pyelonephritis

**DOI:** 10.1186/s12894-020-00596-4

**Published:** 2020-03-11

**Authors:** Mitsuhiro Tambo, Satoru Taguchi, Yu Nakamura, Takatsugu Okegawa, Hiroshi Fukuhara

**Affiliations:** Department of Urology, Kyorin University School of Medicine, Shikawa 6-20-2, Mitaka, Tokyo, 181-8611 Japan

**Keywords:** Acute pyelonephritis, Upper urinary tract calculi, Sepsis, Procalcitonin, Presepsin

## Abstract

**Background:**

Acute pyelonephritis (APN) with obstructive uropathy often causes sepsis. Recently, sepsis was redefined using the sequential organ failure assessment (SOFA) score, based on the new Sepsis-3 criteria. We investigated predictors for sepsis using this new definition in patients with obstructive APN associated with upper urinary tract calculi.

**Methods:**

We retrospectively evaluated patients who were admitted to our hospital for treatment of obstructive APN associated with upper urinary tract calculi. Blood and urine samples were collected before treatment of obstructive APN. Treatment included adequate antimicrobial therapy and emergency drainage to decompress the renal collecting system. We diagnosed sepsis using the new Sepsis-3 definition. We assessed predictors for sepsis by multivariate logistic regression analysis.

**Results:**

Sixty-one patients were included in this study. Overall, all patients underwent emergency drainage, and 11 (18.0%) patients showed sepsis. There were no significant differences in performance status or comorbidities between sepsis and non-sepsis groups. Platelet count and serum albumin level were significantly lower in the sepsis group than in the non-sepsis group (*p* = 0.001 and *p* = 0.016, respectively). Procalcitonin (PCT) and presepsin (PSEP) levels were significantly higher in the sepsis group than in the non-sepsis group (*p* < 0.001 and *p* < 0.001, respectively). Multivariate analysis showed that PCT elevation (OR = 13.12, *p* = 0.024) and PSEP elevation (OR = 13.13, *p* = 0.044) were independent predictors for sepsis.

**Conclusions:**

Elevation of PCT and PSEP levels before treatment might predict the development of sepsis in patients with obstructive APN.

## Background

Sepsis is a major cause of morbidity and mortality among hospitalized patients, and its reported incidence is increasing [1]. Until recently, sepsis was defined as the presence of two or more positive systemic inflammatory response syndrome (SIRS) criteria and confirmed infection [2]. However, SIRS criteria are limited in that they exhibit inadequate specificity and sensitivity for dysregulated, life-threatening responses [3]. In 2015, sepsis was redefined (Sepsis-3 criteria) to emphasize the role of organ dysfunction, using the sequential organ failure assessment (SOFA) [4].

Urosepsis is commonly caused by obstructive disease of the urinary tract, such as urinary stones, stenosis, and tumor, and affects 20–30% of all septic patients [5]. A Japanese multicenter survey revealed that the rate of urosepsis based on SIRS criteria was 35.4% in patients with obstructive acute pyelonephritis (APN) secondary to urolithiasis [6]. A recent retrospective study showed that SOFA was more useful than SIRS for prediction of mortality in patients with APN associated with upper urinary tract calculi [7]. Most previous studies of sepsis have discussed biomarkers for sepsis and septic shock [8]; to the best of our knowledge, there have been no reports regarding markers for sepsis, based on the new definitions, in patients with obstructive APN. Thus, we investigated the predictors for sepsis based on the new definitions in patients with obstructive APN associated with upper urinary tract calculi.

## Methods

This retrospective study was based on a medical chart review of patients who were admitted to our institution for the treatment of obstructive APN with upper urinary tract calculi between September 2015 and February 2018. The definition of APN and the regimen of antimicrobial treatment that we used were described in our previous report [8]. Patients who developed APN associated with surgical intervention were excluded from the final cohort to eliminate a potential bias. Prior to the initiation of treatment, blood samples were collected for biochemical and biomarker measurements, including procalcitonin (PCT) and presepsin (PSEP). Patients who had severe chronic kidney disease (estimated glomerular filtration rate [eGFR] < 30 mL/min/1.73 m^2^) at baseline were also excluded from the final cohort to minimize the impact of kidney function on PCT and/or PSEP values. The severity of acute kidney injury (AKI) was graded according to the AKI network (AKIN) criteria [9]. Midstream urine and blood culture analyses with antimicrobial susceptibility tests were carried out for all patients. When a responsible bacterium showed resistance to the initial empirical treatment, the antibacterial agent was changed into another one to which the bacterium was susceptible. For the purpose of initial emergency drainage of the renal collecting system, retrograde ureteral stenting was generally carried out for most cases, whereas percutaneous nephrostomy was carried out for those who failed initial treatment or supposedly unfit cases for ureteral stenting (e.g. those who had histories of urinary tract abnormalities or severe hydronephrosis). Detailed procedures for retrograde ureteral stenting and percutaneous nephrostomy were described in our previous report [8].

Sepsis was defined based on the Sepsis-3 criteria. Briefly, patients with suspected infection were evaluated using the quick SOFA (qSOFA) (two or more of the following: respiratory rate ≥ 22/min, altered mental state, and systolic blood pressure ≤ 100 mmHg). Patients with positive for qSOFA and ≥ 2 SOFA score points were diagnosed with sepsis [4]. Performance status was classified in accordance with the Eastern Cooprative Oncology Group performance status classification. Cardiovascular or neurologic diseases and immunocompromised status were described in our previous report [8]. Risk stratification of comorbidity was classified using the Charlson Comorbidity Index [10]. The severity of hydronephrosis was categorized as low-grade and high-grade, as reported previously [8]. This study was approved by the Faculty of Medicine Research Ethics Committee, Kyorin University (approval No. H30–196). The review board waived the requirement for consent due to the retrospective nature of the study.

### Statistical analysis

The variables were compared among groups using the Mann–Whitney U-test and Kruskal-Wallis test. The independence of categorical data was estimated by the chi-squared test or Fisher’s exact test. Continuous variables (age, serum creatinine level, leukocyte counts, C-reactive protein (CRP) level, platelet count, serum albumin level, PCT level, and PSEP level) were used to divide the patients into two groups by receiver operating characteristic curve analysis. Independent predictors of sepsis were determined using logistic regression analysis. Differences with *p* < 0.05 were considered to be statistically significant. Statistical analyses were conducted using SPSS software (version 18.0, IBM Corp., Armonk, NY, USA).

## Results

This study included 61 patients. Median patient age was 64 years, 24 patients were male and 37 patients were female. In total, five patients (8.2%) exhibited poor performance status (2–4). Of the 61 patients, there were 16, eight, six, three, and two with diabetes mellitus, cardiovascular diseases, neurologic diseases, immunocompromised status, and urinary tract abnormalities, respectively. Median serum creatinine was 1.22 mg/dL. Overall, 41 patients (67.2%) showed a large shift in leukocyte count (< 4000 or > 12,000/μL). Twenty and 19 patients exhibited reduced platelet count (< 150,000/μL) and reduced serum albumin level (< 3.1 g/dL), respectively. Median PCT and PSEP levels were 1.49 ng/mL and 445 pg/mL, respectively. PCT and PSEP levels increased significantly with the severity of AKI (Table 1). In total, 54 (88.5%) and 22 patients (36.1%) showed bacterial growth in midstream urine culture and blood culture, respectively.

**Table 1:** PCT and PSEP stratified by AKI grade according to the AKIN criteria

Variables	AKI grade				*P*-value
	Grade 0 (*n* = 20)	Grade 1 (*n* = 34)	Grade 2 (*n* = 3)	Grade 3 (*n* = 4)	
PCT, median (ng/mL)	0.40	1.89	84.45	113.46	0.015
PSEP, median (pg/mL)	335	528	835	740	0.001

*PCT* Procalcitonin, *PSEP* Presepsin, *AKI* Acute kidney injury, *AKIN* Acute kidney injury network

Most stones were located in the ureter (90.2%). The median longest and shortest diameters of stones were 8.0 mm and 5.0 mm, respectively. Overall, 22 patients (36.1%) had high-grade hydronephrosis. Fifty-six (91.8%) and five patients (8.2%) underwent indwelling ureteral stenting and nephrostomy, respectively, to achieve decompression of the collecting system. The median duration from initial treatment to decompression of the collecting system was 1.0 days.

**Fig. 1:**
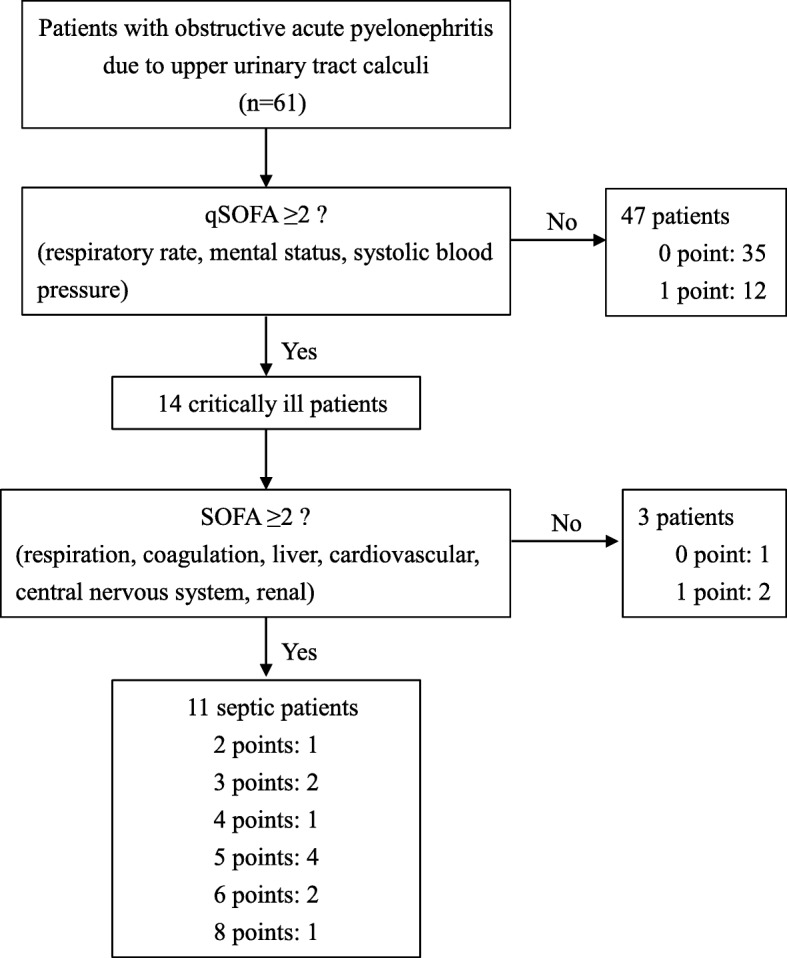
Flow chart representing the diagnostic process of sepsis according to the Sepsis-3 criteria

In total, 11 patients (18.0%) were positive for sepsis, using the Sepsis-3 criteria (Fig. 1). One patient (1.6%) died of the infection. Age did not significantly differ between sepsis and non-sepsis groups (*p* = 0.209). Performance status and underlying diseases did not significantly differ between sepsis and non-sepsis groups. With respect to laboratory data before treatment, platelet count and serum albumin level were significantly lower in the sepsis group than in the non-sepsis group (*p* = 0.001 and *p* = 0.016, respectively). However, inflammatory markers (leukocyte count and CRP level) and serum creatinine did not significantly differ between sepsis and non-sepsis groups (*p* = 0.073, *p* = 0.586, and *p* = 0.058, respectively). PCT and PSEP levels were significantly higher in the sepsis group than in the non-sepsis group (*p* < 0.001 and *p* < 0.001, respectively). There were no significant differences in stone characteristics between the sepsis and non-sepsis groups (Table 2).

**Table 2:** Characteristics of patients with and without sepsis

Variables			Sepsis group		Non-sepsis group		*P*-value
			n	%	n	%	
Patients			11		50		
Age (yr)		Median [IQR]	73 [55–77]		61 [52–76]		0.209
Gender		Male	3	27.3	21	42.0	
		Female	8	72.7	29	58.0	0.365
Performance status		0, 1	9	81.8	47	94.0	
		2, 3	2	18.2	3	6.0	0.182
Diabetes mellitus		Negative	8	72.7	37	74.0	
		Positive	3	27.3	13	26.0	0.931
Cardiovascular or neurologic disease		Negative	7	63.6	42	84.0	
		Positive	4	36.4	8	16.0	0.124
Immunocompromised status		Negative	10	90.9	48	96.0	
		Positive	1	9.1	2	4.0	0.480
CCI scores		0–2	8	72.7	47	94.0	
		≥3	3	27.3	3	6.0	0.066
Serum creatinine (mg/dL)		Median [IQR]	1.50 [1.22–2.14]		1.17 [0.78–1.70]		0.058
Leukocyte counts (×10^3^/μL)		Median [IQR]	18.3 [10.0–22.3]		12.8 [10.1–15.5]		0.073
C-reactive protein (mg/dL)		Median [IQR]	14.17 [4.84–24.51]		10.72 [6.66–16.34]		0.586
Platelet counts (× 10^4^/μL)		Median [IQR]	10.1 [6.1–14.8]		19.2 [15.5–26.9]		0.001
Serum albumin (g/dL)		Median [IQR]	3.0 [2.6–3.5]		3.6 [3.2–4.0]		0.016
PCT (ng/mL)		Median [IQR]	31.57 [1.83–134.40]		0.54 [0.14–4.86]		< 0.001
PSEP (pg/mL)		Median [IQR]	1080 [696–1550]		387 [313–558]		< 0.001
Midstream urine culture		Negative	0	0.0	7	14.0	
		Positive	11	100.0	43	86.0	0.187
Blood culture		Negative	1	9.1	38	76.0	
		Positive	10	90.9	12	24.0	< 0.001
Hydronephrosis		Low-grade	7	63.6	32	64.0	
		High-grade	4	36.4	18	36.0	0.982
Laterality of stone		Right	5	45.5	25	50.0	
		Left	6	54.5	25	50.0	0.785
Position of stone		Renal pelvis or PUJ	2	18.2	4	8.0	
		Upper ureter	6	54.5	29	58.0	
		Mid ureter	1	9.1	8	16.0	
		Lower ureter	2	18.2	9	18.0	0.738
Size of stone (mm)	Longest diameter	Median [IQR]	7.0 [5.0–12.0]		8.0 [6.0–10.0]		0.806
	Shortest diameter	Median [IQR]	4.0 [3.0–8.0]		5.0 [4.0–7.0]		0.842
Drainage		Ureteral stent	10	90.9	46	92.0	
		Nephrostomy	1	9.1	4	8.0	0.905

*CCI* Charlson Comorbidity Index, *PCT* Procalcitonin, *PSEP* Presepsin, *PUJ* Pelvic ureteral junction

Multivariate logistic regression analysis showed that elevated PCT and PSEP levels were predictors for sepsis in patients who had obstructive APN associated with upper urinary tract calculi (Table 3).

**Table 3:** Odds ratio for sepsis in relation to various factors by logistic regression analysis

Variables		Univariate			Multivariate		
		OR	95% CI	*p*-value	OR	95% CI	*p*-value
Age (yr)	64≥	Ref					
	64<	3.13	(0.74–13.20)	0.120			
Gender	Male	Ref					
	Female	1.93	(0.46–8.16)	0.370			
Performance status	0, 1	Ref					
	2, 3	3.48	(0.51–23.89)	0.204			
Diabetes mellitus	Negative	Ref					
	Positive	1.07	(0.25–4.64)	0.930			
Cardiovascular or neurologic disease	Negative	Ref					
	Positive	3.00	(0.71–12.69)	0.136			
Immunocompromised status	Negative	Ref					
	Positive	2.40	(0.20–29.10)	0.492			
History of urinary tract abnormalities	Negative	Ref					
	Positive	1.21	(0.15–9.82)	0.860			
CCI scores	0–2	Ref					
	≥3	5.87	(1.00–34.39)	0.050			
Serum creatinine (mg/dL)	1.26≥	Ref					
	1.26<	3.39	(0.80–14.32)	0.096			
Leukocyte counts (×10^3^/μL)	13.1≥	Ref					
	13.1<	3.13	(0.74–13.20)	0.120			
C-reactive protein (mg/dL)	10.83≥	Ref					
	10.83<	1.20	(0.32–4.45)	0.785			
Platelet counts (×10^4^/μL)	15.0≤	Ref			Ref		
	15.0>	15.95	(3.00–84.92)	0.001	3.23	(0.39–26.64)	0.276
Serum albumin (g/dL)	3.1≤	Ref			Ref		
	3.1>	5.54	(1.38–22.24)	0.016	1.21	(0.15–9.82)	0.860
PCT (ng/mL)	23.55≥	Ref			Ref		
	23.55<	30.67	(5.75–163.67)	< 0.001	13.12	(1.41–122.03)	0.024
PSEP (pg/mL)	515≥	Ref			Ref		
	515<	23.33	(2.73–198.87)	0.004	13.13	(1.07–161.45)	0.044

*CCI* Charlson Comorbidity Index, *PCT* Procalcitonin, *PSEP* Presepsin, *OR* Odds ratio, *CI* Confidence interval

## Discussion

The present study showed that elevated PCT and PSEP levels before treatment might be independent predictors for sepsis, based on the Sepsis-3 criteria, in patients with obstructive APN. Obstructive APN can be lethal, and a recent survey revealed a mortality rate of 2.3% [6]. Based on the new definition, a diagnosis of sepsis is associated [11]. Thus, patients who have obstructive APN and who have elevated PCT and PSEP levels before treatment might exhibit a risk of mortality. However, in adult patients with uncomplicated APN, Cleassens et al. reported that the performance of PSEP level for prediction of positive blood culture (area under the receiver operating characteristic curve [AUC] = 0.63) was similar to that of CRP level (AUC = 0.64) and less accurate than that of PCT level (AUC = 0.78, *p* < 0.001) [12]. The limitations of the study by Cleassens et al. included a low rate of bacterial detection in blood culture of sepsis patients, and low rate of sepsis in patients with uncomplicated APN.

A recent meta-analysis showed that PCT and PSEP levels had moderate diagnostic value for sepsis [13]. However, most previous studies reported that patients had SIRS and/or severe sepsis/septic shock. In addition, in a clinical study of sepsis based on the new definitions, multivariate analysis showed that patients who were admitted to the intensive care unit with high PSEP and PCT levels had significantly higher risk of sepsis, compared with that of patients with low PSEP and PCT levels [14]. The limitations of that study included the use of SIRS criteria for initial screening.

PCT is a prohormone of calcitonin; under normal conditions, it is only produced in C-cells of the thyroid gland. During infection, PCT can be produced by several cell types and many organs in response to pro-inflammatory cytokines (e.g., tumor necrosis factor-α and interleukin-6). Because it shows an early increase during infection, PCT is a useful biomarker for infection [15]. PSEP is the soluble form of CD14, a membrane-based receptor that is expressed by macrophages and monocytes. PSEP is a complex product of CD14 cleavage that is released into the general circulation by proteolysis and exocytosis, which occur following bacterial antigen binding. PSEP levels are presumed to increase during early stages of bacterial infections, and may depend on the intensity of innate immune induction [16]. In an animal model of sepsis, PSEP in the blood was detected within 2 h and peaked after 3 h, while PCT was elevated within 3–6 h and peaked at 6–8 h [17]. In contrast, elevated CRP levels have been found within 6–8 h of infection and peak after 36–50 h [18].

There were several limitations in this study. First, patients with severe chronic kidney disease (GFR < 30 mL/min/1.73 m^2^) were excluded from analysis. PSEP levels inversely correlate with GFR, and PSEP levels in patients with GFR < 30 mL/min/1.73 m^2^ were reportedly significantly higher than those in patients with GFR ≥ 30 mL/min/1.73 m^2^ [19]. The evaluation of PSEP levels in patients with chronic kidney disease thus requires special considerations, such that the present results may not be generalizable to those patients. In the present study, three patients with GFR < 30 mL/min/1.73 m^2^ demonstrated PSEP levels of 1150 pg/mL, 889 pg/mL, and 3620 pg/mL, respectively; all were negative for sepsis. Second, levels of PCT and PSEP could be influenced by acute kidney injury (AKI). Recently, a retrospective study in patients with AKI showed that different thresholds of PSEP and PCT levels might be useful markers of bacterial infections [20]. Another study demonstrated that the accuracy of sepsis diagnosis based on PCT level was significantly higher than that based on PSEP level in patients with severe AKI [21]. However, the values for cutoff levels, depending on renal function, remain controversial. Finally, our results were limited to a small number of enrolled patients and were retrospectively collected. Further studies should include larger prospective cohorts.

## Conclusions

Elevated PCT and PSEP levels before treatment might predict the development of sepsis in patients with obstructive APN.

## Data Availability

The datasets used and/or analysed during the current study are available from the corresponding author on reasonable request.

## Abbreviations

AKI: Acute kidney injury
AKIN: Acute kidney injury network
APN: Acute pyelonephritis
CRP: C-reactive protein
PCT: Procalcitonin
PSEP: Presepsin
qSOFA: quick SOFA
SIRS: Systemic inflammatory response syndrome
SOFA: Sequential organ failure assessment
